# Late preterm infants’ motor development until term age

**DOI:** 10.6061/clinics/2017(01)04

**Published:** 2017-01

**Authors:** Viviane Martins Santos, Cibelle Kayenne Martins Roberto Formiga, Paulo Roberto Bezerra de Mello, Cléa Rodrigues Leone

**Affiliations:** I Universidade Federal do Mato Grosso, Hospital Universitário Júlio Müller, Cuiabá/MT, Brazil; II Universidade Estadual de Goiás, Faculdade de Educação Física e Fisioterapia, Goiania/GO, Brazil; III Universidade Federal de Mato Grosso, Departamento de Pediatria, Cuiabá/MT, Brazil; IV Universidade de São Paulo, Departamento de Pediatria, São Paulo/SP, Brazil

**Keywords:** Motor Skills, Infant Premature, Child Development, Early Diagnosis, Cohort Studies

## Abstract

**OBJECTIVES:**

To analyze the motor development of late preterm newborn infants (LPI) from birth to term-corrected age using the Test of Infant Motor Performance (TIMP) and to compare the obtained results with those of term infants at birth.

**METHODS:**

Prospective cohort study, 29 late preterm newborn infants were evaluated by the TIMP at birth and every two weeks until term-corrected age. The TIMP was administered to 88 term infants at birth.

**RESULTS:**

The mean TIMP score of late preterm newborn infants was 51.9±5.8 at 34–35 weeks and 62.6±5.2 at 40 weeks. There was a significant increase at 38–39 weeks in the LPI group (*p*<0.05). There were no significant differences in the motor evaluations between term infants at birth and LPI at the equivalent age.

**CONCLUSION:**

The LPI presented a gradual progression of motor development until the term-corrected age, but differences with term infants at birth were not detected.

## INTRODUCTION

Gestational age is one of the main factors that influence maturation and the evolution of neonatal development 1,2. Newborn infants with a gestational age between 34 and 36 6/7 weeks are referred to as “late preterm,” according to the National Institute of Child Health and Human Development (NICHD) in 2005 3; this designation was made in recognition of the physiological and developmental immaturity of this group of infants at birth 2.

At the end of gestation, dramatic changes occur in brainstem development in terms of neuronal origin and proliferation, migration pathways, morphological and neurochemical differentiation, neurotransmitter receptors, neurotransmitters and enzymes, dendritic arborization, spinal formation, synaptogenesis, axonal growth, and myelination 4,5. Thus, the period between 34 and 40 weeks of gestation is considered a critical growth period for the development of many neural structures and connections; moreover, cortical volume increases by 50% during this time. It follows that the occurrence of brain injury during this period might impact late cerebral development 4,6,7. Therefore, this step in neurological development, which should occur before birth, happens during the extra uterine phase in late preterm infants (LPI). This results in additional risks to these newborn infants and the potential for associated clinical manifestations later in life.

Regarding motor development of LPI, a program of developmental physiotherapy was applied to that infants born preterm from term to four months corrected age. The intervention group improved their performance in relation to the nonintervention infants and at four months corrected age they performed similarly to the reference group 8.

Early evaluation of function may facilitate early intervention, thus enabling adequate treatment for the detected problems, which may reduce future motor, school, and psychosocial deficiencies 9,10.

Considering the above mentioned risks on LPI, specifically those regarding motor development deviations, the early identification of motor injury is essential to the timely provision of appropriate support and interventions.

## PURPOSE

This study aimed to analyze the motor development of LPI from birth to term-corrected age using the Test of Infant Motor Performance (TIMP) and to compare the obtained results with those of term infants (TI) at birth.

## METHODS

A prospective and observational cohort study was developed in four hospitals accredited by the public health system of Cuiabá, Mato Grosso/Brazil. The sample included newborns that fulfilled the inclusion criteria during the period of July 2012 to December 2013. This research project was approved by the Research Ethics Committee of the Júlio Müller University Hospital, Federal University of Mato Grosso, under the protocol number 938/2010; and by the Commission for Analysis of Research Projects, Clinical Director Office, Hospital das Clínicas da Faculdade de Medicina da Universidade de São Paulo. The mothers or tutors were informed of the research objective and, after agreeing to participate, signed the informed consent form.

### Participants

The sample of this study consisted of 29 LPI and 88 TI at birth. The newborns were selected according to the following inclusion criteria: gestational age (GA) at birth between 34 and 36 6/7 weeks for the LPI group and 38 to 40 6/7 weeks for the TI group; birth weight between the 10^th^ and 90^th^ percentile according to the fetal growth curve described by Alexander et al. (1996) 11; LPI with cranial ultrasound (US) in the first 2 weeks after birth with grade 0 or I intracranial hemorrhage.

The following exclusion criteria were used: children whose mother showed a positive serology for congenital infections during gestation or at delivery, had a history of using illegal drugs, and/or was under 16 years of age; genetic malformations or chromosomal abnormalities; prolonged (a week or longer) use of sedatives or neuromuscular blockers; need for surgical procedures until 40 weeks of corrected GA; oxygen dependency with 40 weeks of corrected GA; brachial or medullary plexus injury and/or occurrence of necrotizing enterocolitis.

The TI with intercurrences, which could have led to alterations in cranial US, were excluded from this study. The gestational age range for that group was limited to reduce variability of the gestational age for this reference group.

GA was based on the US performed before the 20^th^ week of gestation. After GA was determined, the newborns were divided into two groups according to their GA at birth: LPI group and TI group. For the LPI group, motor development was assessed through the Test of Infant Motor Performance(TIMP) 12,13,14 every two weeks until term-corrected age (40 weeks); a cranial US was performed during the first two weeks of age; weight, length, and head circumference (HC) measurements were also taken. In TI group, the motor development was measured through one evaluation by the TIMP after 24 hours of life.

The Test of Motor Infant Performance (TIMP) is a motor scale that assesses motor and postural control and is considered appropriate for evaluating the motor development of preterm-born children with a post-conception gestational age of 32 weeks until four months past term age. In addition, it can quantitatively assess motor development, which allows the evaluation of motor development after discharge 12,13,14,15.

The Test of Motor Infant Performance (TIMP) tool version, 5.1 15 was used for evaluation of motor development, as well as the Test User’s Manual version 2.0 16, the Test User’s Manual version 3.0 for the TIMP version 5.0 17, the Self-instructional CD v.4 18, and the Percentile Rank Score Sheet 19. These materials were acquired through the TIMP website (http://www.thetimp.com), which has the authorial rights for these tests.

The TIMP, version 5.1 (in Portuguese), consists of 42 items: 13 observed and 29 elicited. For the observed items, a score of 0 is given if the item is absent, and 1 if it is present. The evaluated observed or tested items are as follows: head orientation, body alignment, distal leg movements, antigravity control, and response to auditory and visual stimuli. Each item has its own scale, which varies from 1–6. The score of each item is added to yield the overall score. According to the manual, the TIMP may be used on children born preterm and full-term, and requires an average time of 33 minutes to administer and score 16,17,18.

Motor evaluations were performed by two physiotherapists trained via the Self-instructional CD v.4 19, Test User’s Manual version 2.0 17, and the Test User’s Manual version 3.0 for the TIMP version 5.0 18,20,21 . The principal investigator received training and conducted the video analysis of the TIMP application in research. Data were collected and media stored for future analysis. The inter-observations concordance test was conducted with videos and the Kappa coefficient 0.96.

The motor evaluations of the LPI were conducted after stabilization and/or at 24 hours of age, and were re-evaluated fortnightly, on the day that corresponded to the first evaluation, until term-corrected age (40 weeks). The term newborns were evaluated between 24 and 48 hours after birth.

The evaluation was conducted in a quiet environment, with adequate lighting and controlled ambient temperature. The newborns wore minimal cloths to prevent movement restriction, were in States 3, 4, or 5, according to Brazelton criteria 22, and between feeding periods. The evaluations were conducted in the presence of the mother or tutor, and were recorded using a film camera (DCR-SX44/S 4GB Handy Cam, 60x Optical Zoom with a 2.4” Touch Screen monitor – Sony, China).

The investigator placed the baby on a firm surface in the neonatal unit, or in the hospital room or home, and removed the baby’s diaper and excess cloths. The camera was positioned beside the evaluator. Then, the vital signs were evaluated and spontaneous activities were observed (observed items from the TIMP). Following this, the evaluations of the elicited items from the TIMP were conducted (items 14–42).

Unless otherwise stated, visual and/or verbal stimuli were used to elicit responses from the baby. A maximum of 3 attempts were allowed for each item, depending on the baby’s response, level of alertness, and tolerance; however, one attempt was usually sufficient, preventing the induction of unnecessary tiredness in the baby. After finishing the test, the videos were stored on a digital media for later evaluation, and an identification code was given to the child.

During the video analysis, all the annotations and documentations regarding the responses from the babies were registered on the form provided by the TIMP, version 5.1 in Portuguese 16. After documenting the initial observations, the investigator continued to watch the baby and noted any additional occurrences of the observed items during the remaining part of the test. Based on these observations, the best response from the baby was ultimately scored.

In cases when a baby did not perform a given response, the closest response was scored. If an evaluation was interrupted, the remaining items were completed in a second session conducted within the following 24 hours.

The following data were obtained from each newborn’s record: mother’s characteristics (age, use of medications, presence of intercurrences, socioeconomic level, birth conditions, type of delivery and anesthesia); newborn characteristics (Apgar score at 5 minutes, neonatal resuscitation, gender, birth weight, length, and head circumference – HC); neonatal evolution of the LPI until term-correct age (intercurrences, weight, length, head circumference and motor evaluation).

Statistical analysis was used for sample calculation, in which the means and standard deviations of the TIMP scores obtained from infants of various gestational ages measured in Campbell (2006) 15 were used as a reference. Therefore, for a standard deviation of 15.0 and the ability to detect variations between score averages of at least 11, obtained in a pilot study performed by the authors, a power of 80% and a significance level of 5% were calculated as necessary 29 children in each group of the study. To determine sample size, the STATCALC from the software Epi Info version 7.0 was used. Regarding group composition, a proportion of 1 case per 3 controls (TI) was maintained.

For all the analyses, a significance level of *p*<0.05 was used. The programs Excel 2007, SPSS version 20.0, and Epi Info version 7.0 were used. The Kolmogorov-Smirnov test and Levene’s test were used for verifying normality and homoscedasticity, respectively. Subsequently, the Student’s t-test for two paired samples and two independent samples, and multiple linear regression were used to identify predictor variables for the LPI motor performance. All variables that showed a *p*<0.20 were included in the multiple linear regression and sequentially removed until only significant variables remained. To compare qualitative variables the Fisher's exact test and the Chi-square test were used.

## RESULTS

A total of 112 TI and 50 LPI were evaluated. Data from 23 TI were discarded because the infants were evaluated earlier than 24 hours after birth, and data from 1 were excluded because the infant had a congenital hip deformity (detected during the exam and subsequently confirmed). Data from 21 LPI were excluded: 3 infants were classified as small for GA; 1 progressed to death; and 17 did not undergo a proper evaluation as defined by the established protocol. Thus, the final sample of the study was composed of 29 LPI and 88 TI.

Table 1 describes the characteristics of the newborns in the groups. As expected, the LPI group presented at birth with a statistically lower GA, weight, length, and HC relative to the TI group (*p*<0.001).

A progressive increase in weight was observed over time in the LPI group according to the corrected gestational age (CGA). At 40 weeks CGA, this variable was within normal limits as established by Alexander 11.

**Figure 1:**
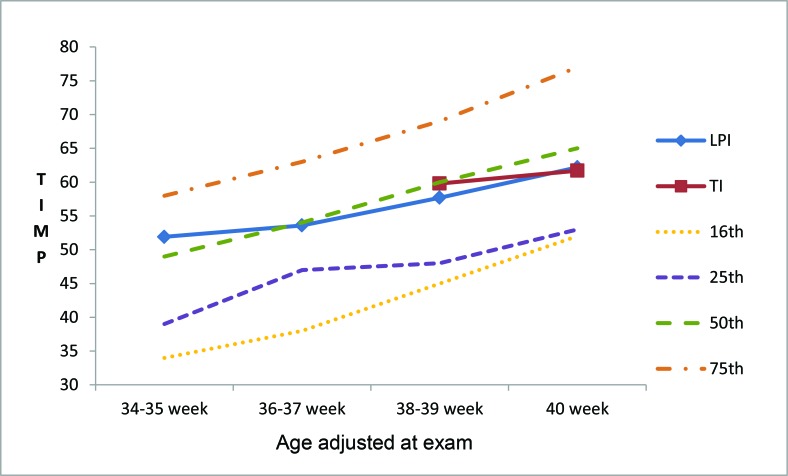
Progression of total Test of Infant Motor Performance (TIMP) score in percentile, according to the corrected gestational age of late preterm newborn infants (LPI) and term infants(TI) at birth. Source: Adapted from TIMP Percentile Rank Standards 2004 19.

The motor progression in LPI, as analyzed by the TIMP, according to CGA is represented in Figure 1; there was a significant increase in the scores of the elicited items and in total TIMP score during weeks 38–39 (*p*<0.05). As shown in Table 2, the observed items did not show significant differences according to the evolution of the CGA of the LPI. However, significant increases in the total score and the mean scores of the elicited items were observed in LPI from 38–39 weeks until 40 weeks CGA. The TIMP scores did not significantly differ between LPI at term age and TI at birth (Table 3).

After classifying motor performance as normal or delayed according to the values established by Campbell 15, we verified that 20 (69%) LPI were classified as normal at 38–39 weeks CGA, and 13 (87%) at 40 weeks. For the TI at birth, 54 (82%) were classified as normal at 38–39 weeks and 21(96%) at 40 weeks. When comparing the children classified as normal, there was no evidence of differences between the LPI groups, with CGA equivalent to term, and TI at 38–39 or at 40 weeks in TIMP scores.

Of the 29 LPI, a cranial US within normal limits was observed in 23 (79.3%); a unilateral intracranial hemorrhage grade I was found in 2 (6.9%); and a bilateral intracranial hemorrhage grade I was observed in 4 (13.8%).

Multiple linear regression revealed that maternal age and HC at birth predicted motor performance scores on the TIMP in LPI at the age equivalent to term (Table 4).

## DISCUSSION

As a result from the immaturity of their systems at birth, especially that of the central nervous system (CNS), LPI are more likely to present with developmental disorders in the period after birth. Evaluating the motor development of these newborns after birth may facilitate the detection of developmental deviations and the timely administration of interventions. In this study, we evaluated the progression of motor development in LPI until term-corrected age. The results indicated a gradual increase in TIMP scores, ultimately culminating in scores that did not differ from those of the TI at birth and that were very close to the 50^th^ percentile, based on the reference established by Campbell 15.

The fact that we did not detect differences between groups at term might be a consequence of the sample selection based on restricted inclusion criteria, which defined a low risk population. In support of this hypothesis is the fact that the LPI presented a growth within the normal range by the Alexander 11. Moreover, cranial US was normal in approximately 80% of LPI, and a mild intracranial hemorrhage was detected in the remaining LPI.

The reports on neurological and health development in extremely preterm newborns (GA<28 weeks) are reasonably well documented; however, little is known about LPI (34≤GA<37 weeks). The term “LPI” emphasizes the vulnerability of these children due to the lack of full growth and development, particularly in the CNS. Understanding of the cellular and molecular mechanisms underlying brain vulnerability in LPI is necessary for the planning of relevant interventions and therapies 23.

The exact sequence of gray matter maturation is presently unknown, and the order is defined by the embryonic (and phylogenetic) pattern of structural development before the association with the cortical areas 23.

Given the continuous and gradual development between 34 and 40 weeks GA, the LPI in the present study were at a lower risk for neurological injury, mainly because of the good conditions at birth and the lower degree of immaturity in relation to the corresponding term; this low risk was additionally verified by the results of the cranial ultrasound. The development of these children birth may therefore have been intensely influenced by their environment. Such circumstances may explain the observed pattern of linear motor development in the LPI: a significant increase in TIMP scores from 38–39 weeks CGA (Table 2) and the lack of significant differences from TI at term (Table 3); these findings suggest a physiologically evolving process.

On the other hand, it should be noted that the TI were evaluated 24 hours after birth, and might have consequently been undergoing the perinatal adaptation period. From this perspective, this situation could be a limitation of this study, as this influence could be excluded if this evaluation had been repeated after 72 hours of age, when the newborns had already adapted to the extra uterine conditions. This was not possible because newborns do not typically remain in the public health system for an extended period after birth in Cuiab�.

In 2010, Raniero 24 used the TIMP to assess the motor performance of 12 healthy preterm newborns with a average GA of 33.6 weeks, and monitored them until 4 months CGA; results showed that the motor ability increased in all ages, and the rate of motor acquisition was higher from 0–1 month than from 3–4 months. These results are in agreement with the results of the present study.

Comparing the average TIMP total score of the LPI in this study (Table 2) with the means established by Campbell 15, we observed that the scores found at the evaluated corrected ages were similar to those established in this study, close to the 50^th^ percentile (Figure 1). The standardization of the TIMP results by Campbell was conducted from 34 weeks after conception until 17 weeks CGA, where the means were determined in 2-week intervals. Although there is no standardization of TIMP scores for the Brazilian population, the American standardization was used because it was conducted with 990 infants representative of the diverse ethnicities and regions of the USA; in addition, the means and standard deviation were calculated for each one of the twelve groups according to age and final development of the TIMP version 5 15,16.

When examining total TIMP scores of healthy preterm newborns with a mean GA at birth of 33.6 weeks, Raniero 24 also verified that until 3 months CGA, the preterm newborns presented values lower than those expected by Campbell 15, but without significant differences.

Although LPI spend an important phase of CNS development in the intrauterine environment, this process continues after birth through the myelination of the white substance, neuronal and gyrus differentiation and increase of the gray matter volume 25. Thus, in the present study a longitudinal follow up until the age equivalent to term revealed that the development of these LPI without severe neurological injuries, was compatible with the motor evaluation of TI at birth (38–39 as well as 40 weeks) (Table 3).

For the purposes of classifying total motor performance score, newborns who score below -0.5 SD (standard deviation) should be classified as delayed and monitored for a longer period of time 15. Although the mean TIMP total score of the LPI at the term age in our results did not differ from that of TI at birth and was close to the 50^th^ percentile, 31% of LPI and 18.27% of TI obtained a total score lower than -0.5SD at 38-39 weeks. These subjects were classified as delayed and therefore should be monitored for a longer period.

A multiple linear regression analysis verified that maternal age and HC at birth were predictors of motor development in LPI at the age equivalent to term (Table 4). These results emphasize the importance of HC at birth as a possible indicator of motor development until full term in LPI. This positive association might reflect CNS immaturity and lower CNS volume in LPI at birth, suggesting that an increase in cerebellar and cerebral cortical volume will still occur 6.

However, the influence of maternal age on motor performance is controversial. In a linear regression analysis, maternal age was identified as a negative predictor of the TIMP total score at the age equivalent to term. A negative influence of maternal age on motor development was also described by Borba 25, in which motor performance was evaluated using the Alberta Infant Motor Scale (AIMS) over the course of four months.

It may be that maternal age cannot be discussed as a single or an isolated factor that influences motor development in a general way. Other conditions should be considered including family income, child care, whether the child attends a nursery, the number of offspring or children at home, maternal and paternal education level, maternal marital status, total number of people at home.

Our observation that the cranial US were within normal parameters for 79.3% of LPI is consistent with evidence suggesting that the increase in GA decreases the risk of intraventricular hemorrhage and periventricular leukomalacia. However, LPI are not free of other complications associated with the risk of brain damage, which may not be associated with motor disabilities, such as learning difficulties, suggesting that injuries in other cortical areas may occur 7.

The data from the present study demonstrate that LPI with low risk of neurological injury may exhibit, at term age, a motor performance that does not significantly differ from that of TI at birth. Although the mean TIMP score did not significantly differ between the groups, we detected a motor development delay in some LPI and term infants. These results emphasize the need for monitoring motor development of these newborns from birth until at least term corrected age.

## AUTHOR CONTRIBUTIONS

Santos VM main researcher, was responsible for the survey of literature, study design, data collection and analysis, manuscript writing and approval of the final version of the manuscript. Formiga CK research collaborator, was responsible for data analysis and interpretation of data, critical revision of the manuscript and approval of the final version of the manuscript. Mello PR conceived and designed the study, was responsible for the literature survey, acquisition of data, critical revision of the manuscript, approval of the final version of the manuscript and submission procedures of the manuscript. Leone CR was responsible for the research orientation, conception and design of the study, analysis and interpretation of data, manuscript draft, critical revision of the manuscript and approval of the final version of the manuscript.

## Figures and Tables

**Table 1:** Characteristics of the newborns

Characteristics of the newborn	LP (n=29)	TI (n=88)	*p*
Male (%)	17 (58.6)	37 (42.0)	0.122^A^
Cesarean section (%)	15(51.7)	54(61.4)	0.362^A^
Apgar 5^th^ min > 6 (%)	29(100)	88(100)	-
Gestational age (weeks)	35.4±0.6	38.9±0.8	<0.001^B^
Birth weight (g)	2681±286	3328±287	<0.001^B^
Length (cm)	45.5±1.8	48.4±1.7	<0.001^B^
HC (cm)	32.6±1.6	34.8±1.4	<0.001^B^

A – Chi-square test

B – Student’s t-test

**Table 2:** Progression of the Test of Infant Motor Performance scores in late preterm newborn infants according to the corrected gestational age

	34–35 weeks n=14	36–37 weeks n=14	38–39 weeks n=29	40 weeks n=15
Observed Items	10.7±0.6	10.7±0.8	10.8±0.7	11.1±0.6
Elicited Items	41.2±5.2	42.9±6.2	46.9±6.9*	51.5±5.0*
Total Score	51.9±5.8	53.6±6.4	57.7±7.3*	62.6±5.2*

Student’s t-test *- *p*<0.05

Elicited items and total score: 38–39 weeks x 36–37 weeks; 40 weeks x 38–39 weeks: *p*<0.05

**Table 3:** Test of Infant Motor Performance scores of late preterm newborn infants according to corrected age and of term infants at birth

	38–39 weeks		40 weeks	
	LPI (n=29)	TI (n=66)	LPI (n=15)	TI (n=22)
Observed items	10.8±0.7	10.8±0.7	11.1±0.6	10.8±1.1
Elicited items	47.0±6.9	49.1±6.2	51.1±5.0	50.9±4.8
Total score	57.7±7.3	59.8±6.4	62.6±5.2	61.7±5.0

*p*>0.05 for all comparisons

**Table 4:** Predictors of the Test of Infant Motor Performance score in late preterm newborn infants at the age equivalent to term

Variables	Coefficients	Standard Error	t	*p*
Maternal age	-0.533	0.187	-2.849	<0.05*
Head circumference at birth	2.213	0.618	3.580	<0.001†

* *p*<0.05;

† *p*<0.001
